# Preparation and Characterization of Bio-Based Freshness Indicator Labels Loaded with Natural Pigments with High Stability and Sensitivity

**DOI:** 10.3390/foods13244049

**Published:** 2024-12-15

**Authors:** Yinglin Tan, Xiao Liu, Zhi Cheng, Qiping Zhan, Liyan Zhao

**Affiliations:** 1 College of Food Science and Technology, Nanjing Agricultural University, Nanjing 210095, China; tanyinglin@stu.njau.edu.cn (Y.T.); liuxiao970411@stu.njau.edu.cn (X.L.); chengzhi@stu.njau.edu.cn (Z.C.); qipingzhan@njau.edu.cn (Q.Z.); 2 Sanya Institute, Nanjing Agricultural University, Sanya 572024, China

**Keywords:** natural color, stability, sensitivity, indicator label, freshness

## Abstract

Freshness indicator labels are crucial for food quality monitoring. However, existing labels often lack stability and sensitivity. This study aims to develop a safe freshness indicator label with high stability and sensitivity. By evaluating the pH response characteristics and stability of four natural pigments, purple potato anthocyanin (PA) was identified as having the best color properties. Mixing the more stable alizarin (AL) with PA improved the stability of the pigment solution without reducing sensitivity. These film labels are prepared with three natural biomolecules and polymers that are a two-by-two composite of them: soybean isolate protein, acacia bean gum, and sodium alginate. Through comparisons of ammonia response, color stability, water solubility, and mechanical properties, the soy protein isolate and locust bean gum composite were determined to be the optimal substrate system. The label of soybean protein isolate and locust bean gum was initially applied to the freshness identification of shrimp and chicken. The results show that the label can effectively respond to the spoilage of aquatic products and meat products and has great application potential in the field of food packaging.

## 1. Introduction

Aquatic and meat products are highly susceptible to spoilage, and their freshness is directly related to consumer health and the economic value of these products. During the spoilage process of aquatic products, the metabolic activities dominated by spoilage microorganisms such as Pseudomonas spp. produce volatile basic nitrogen (TVB-N) composed of nitrogen compounds like ammonia, dimethylamine, and trimethylamine. Meat products, which have high protein content, are prone to deterioration due to enzyme-induced protein hydrolysis and microbial metabolism. Thermophilic aerobic bacteria and coliforms are the main bacterial strains leading to the spoilage of meats, producing amine compounds and hydrogen sulfide gas [1,2]. These gases cause changes in the pH of both the environment inside the package and the food itself. Therefore, pH is an important indicator for assessing the freshness of aquatic and meat products [3]. Based on this principle, the development of indicator labels with pH-sensitive pigments has become an effective method for real-time monitoring of the freshness of perishable foods [4,5]. However, existing freshness indicators still face significant challenges in terms of stability, sensitivity, and safety, which limit their practical applications.

In recent years, researchers have increasingly turned to natural ingredients such as anthocyanins, curcumin (CU), alizarin (AL), and beetroot red pigments to replace artificial colors used in food packaging [6]. Due to their wide availability, non-toxic nature, and environmental friendliness, these natural pigments are ideal color development agents for freshness indicator labels. However, there are significant differences in stability and sensitivity among various natural pigments [7]. For instance, some natural pigments are prone to degradation or discoloration under light, temperature changes, and pH fluctuations, which affect their reliability in practical applications [8]. Among them, anthocyanin pigments generally have excellent pH indicator performance, but their stability is also greatly affected by the environment. Studies have shown that mixing two different types of pigments can result in more pronounced color changes, as well as greater color stability and correlation with food freshness [9,10]. Therefore, screening natural pigments with high stability and sensitivity, and further enhancing these properties, has become a key issue in the development of high-performance freshness indicator labels.

The choice of label substrate also significantly impacts the performance of freshness indicators, including sensitivity to lower pH values, response time to pH changes, repeatability, and reversibility [11,12]. Common film-forming substrates include proteins and polysaccharides, which have become viable alternatives to synthetic polymers in the food packaging industry [13]. Soy protein isolate is one of the most widely used vegetable proteins. Soy protein film has better hydrophobic properties than polysaccharide film but suffers from poor mechanical properties. Anionic polysaccharides can bind to anthocyanins through hydrogen bonding and electrostatic interactions, while cationic polysaccharides cannot form specific adsorption with anthocyanin cations [13]. Sodium alginate is an anionic polysaccharide commonly used in membrane production, and locust bean gum is a typical neutral polysaccharide. Therefore, these two polysaccharides were selected as the substrate for the film label. If polysaccharides are used alone to form a film, the overall structure tends to become brittle, and the waterproof ability is poor. Combining different substrates can enhance the mechanical properties and structural complexity of the film [14].

This study aims to develop a freshness indicator label utilizing natural pigments and bio-based materials to enhance its stability and sensitivity. Four natural pigments, including purple potato anthocyanin (PA), blueberry anthocyanin (BA), CU, and AL, were screened to evaluate their pH color properties, visible spectrum, and stability under varying temperatures and light conditions. Using soy protein isolate, locust bean gum, and sodium alginate as substrates, the optimal substrate system was determined by comparing their pH and ammonia response sensitivity, color stability, water solubility, and mechanical properties. Additionally, the selected labels were preliminarily applied to the freshness indication of shrimp and chicken and evaluated. The careful selection and optimization of natural pigments and bio-based materials not only improves the performance of freshness indicator labels but also aligns with the principles of sustainable development [12]. The findings of this study will provide a theoretical foundation and technical support for the development of freshness indicator labels and facilitate their widespread application in the food packaging industry.

## 2. Materials and Methods

### 2.1. Materials

Fresh purple potatoes (Xu purple potato No. 8 variety) originated from Xuzhou Academy of Agricultural Sciences Modern Demonstration Park (Xuzhou, China). BA was purchased from Utop Technology Suzhou Co., Ltd. (Suzhou, China). CU and chymosin were purchased from Shanghai Myriad Biochemical Science and Technology Co., Ltd. (Shanghai, China). α-Amylase (1500 U/g) was purchased from Shanghai McLean Biochemical Technology Co., Ltd. (Shanghai, China). Cellulase (5 × 10^4^ U/g) was purchased from Cangzhou Xiasheng Enzyme Biotechnology Co., Ltd. (Cangzhou, China). Soy protein isolate is sourced from Shanghai Yuanye Biological Technology Co., Ltd. (Shanghai, China). Locust bean gum and alginic acid powder are sourced from Henan Wanbang Chemical Technology Co., Ltd. (Zhengzhou, China). The experimental water used in the experimental process was deionized water, and all other reagents were analytically pure.

### 2.2. Enzymatic Hydrolysis of Ultrasound-Assisted Alcohol Extraction of PA

Based on the method of Sun et al. with some modifications [15]. Firstly, a slicer cut fresh purple potatoes into 3 mm thin slices. The slices were dried in a hot-air drying oven (Shanghai Yiheng Technology Co., Ltd., DHG-9030A, Shanghai, China) at 45 °C until they reached a constant weight. The dried purple potatoes were then crushed and passed through a 60-mesh sieve. A solution was prepared by mixing the purple potato powder with ethanol (65%, *v*/*v*) at a material–liquid ratio of 1:40 (g/mL), and citric acid was added to adjust the pH to 3.0. Cellulase and α-amylase were added to the solution at a mass ratio of 1:1, with a total enzyme amount of 4 mg/g. Parameters of enzymatic hydrolysis at 50 °C for 10 min, ultrasonic cleaning instrument (Guangdong Dongguan Kangshijie Ultrasonic Technology Co., Ltd., PL-S100, Dongguan, China) for 30 min, ultrasonic power of 600 W, frequency of 40 KHZ, temperature of 25 °C, and 4000 r/min centrifugation for 15 min were used. The supernatant was collected and concentrated using a rotary evaporator (Dalong Xingchuang Experimental Instrument (Beijing) Co., Ltd., RE100-Pro, Beijing, China) to obtain the PA extract. The PA powder was freeze-dried, stored at −20 °C, and protected from light at all times.

### 2.3. Screening of Natural Colors

#### 2.3.1. Determination of pH Color Sensitivity of Natural Pigments

In total, 800 mg of freeze-dried PA powder, 150 mg of blueberry anthocyanin powder, 0.5 mg of turmeric powder, and 5 mg of AL powder were weighed. These were dissolved in 100 mL of 65% ethanol to form a pigment solution. The PA solution and AL solution were mixed in ratios of 1:1, 1:3, and 3:1. Three milliliters of the pigment solution were taken, and the pH was adjusted to a range of 2–13 using hydrochloric acid and sodium hydroxide solutions. Photos of the pigment solution were taken to document the color. The visible spectra of the pigment solution were measured using a UV–visible spectrophotometer (Beijing Puyang General Instrument Co., Ltd., TU-1900, Beijing, China) at 400 to 800 nm [16].

#### 2.3.2. Determination of Pigment Stability

The maximum absorption wavelength of the pigment solution was determined by using a UV-Vis spectrophotometer. At the maximum absorption wavelength, the absorbance was determined. The formula for pigment retention was calculated as follows:

(1) $$w\;\left(\%\right)=\frac{A}{A_{0}}\times 100\%$$

where w (%) is the pigment retention rate; A_0_ is the initial absorbance of the pigment solution; and A is the absorbance of the pigment solution after various treatments.

A certain concentration of pigment solution was divided into several equal parts and placed in environments of 4 °C, 25 °C, and 40 °C in the dark for 48 h, as well as in a room temperature environment with sufficient light, and another part in a room temperature environment with no light. The absorbance was measured at 0, 8, 16, 24, 32, 40, and 48 h, and the pigment retention rate was calculated.

### 2.4. Preparation of Indication Labels on Different Substrates

Film labels were prepared using the solution casting method [17]. Initially, 2 g of soy protein isolate, 1 g of locust bean gum, and 1 g of sodium alginate were each added to 80 mL of distilled water containing 3% glycerol. The solution was stirred at 60 °C using a thermostatic magnetic stirring water bath (Changzhou Deke Instrument Manufacturing Co., Ltd., HDK-06, Changzhou, China) until completely dissolved. After cooling to room temperature, 20 mL of PA-AL mixed pigment solution was added and thoroughly stirred. The resulting solution was then defoamed for 5 min using an ultrasonic cleaner. Subsequently, 60 g of the film-forming solution was poured into a 60 mm × 60 mm Petri dish and dried in an oven at 40 °C for 12 h to obtain the dry film. Finally, the dry film was peeled off from the Petri dish and cut into 3 × 1 cm label strips. Composite indicator labels were prepared in the same manner, with substrates combined in a ratio of 1:1. The label samples were named as follows:

(1) SP: soya bean isolate protein indicator label
(2) LB: acacia bean gum indication label
(3) SA: sodium alginate indicator label
(4) SP-LB: soya bean isolate protein–acacia bean gum composite indicator label
(5) SP-SA: soya bean protein–sodium alginate composite indication label
(6) SA-LB: sodium alginate–sophora bean gum composite indication label

### 2.5. Indicator Label Performance Measurement and Substrate Screening

#### 2.5.1. Appearance and Color

The prepared label sample was placed on white paper, and the brightness (L*), red/green (a*), and yellow/blue (b*) of the film label were measured by the colorimeter (Guangdong three Enshi technology Co., Ltd., NR110, Guangdong, China). Each sample was repeated 3 times to calculate the average. The appearance of the label is recorded by taking pictures under constant lighting. The total color difference value calculation formula is as follows:

(2) $$∆E=\sqrt{\left(L^{*}-L_{0}^{*})+(a^{*}-a_{0}^{*})+(b^{*}-b_{0}^{*}\right)}$$

where L*, a*, and b* denote luminance, red-greenness, and yellow-blueness, respectively; *L*_0_, *a*_0_, and *b*_0_ are the color parameters of the standard whiteboard. ΔE > 5 is the color difference that the naked eye can perceive.

#### 2.5.2. Determination of Water Content and Water Solubility

Referring to the method of Zhang et al. [18], the water content and water solubility of the label were determined. The weight of the label sample was recorded as *w*_0_. The labels were baked at 105 °C until a constant weight was achieved, and this weight was recorded as *w*_1_. The labels were then immersed in 30 mL of water for 24 h. The water on the surface of the film labels was blotted with filter paper. The labels were baked again until a constant weight was reached, and this weight was recorded as *w*_2_. The formulas for the calculation of the water content and the water solubility of the samples are as follows:

(3) $$\mathrm{Water}\;\mathrm{content}\;(\%)=\frac{w_{0}-w_{1}}{w_{0}}\times 100$$

(4) $$\mathrm{Water}\;\mathrm{solubility}\;(\%)=\frac{w_{1}-w_{2}}{w_{1}}\times 100$$

#### 2.5.3. pH and Ammonia Sensitivity

The indicator label (3 cm × 1 cm) was immersed in a buffer solution with a pH range of 2–13 until the color stabilized. The colorimeter was used to measure the *L*, *a*, and *b* values of the label, and the ΔE value was calculated according to Formula (2). Here, *L*_0_, *a*_0_, and *b*_0_ were the initial chromaticity values for each label.

The label was then fixed in the center of a cap, which was inverted over a container holding 250 mL of ammonia (4 mmol/L). The color change of the label was recorded at regular intervals. The image feature signal was extracted using Photoshop. The *R*, *G*, and *B* values of 5 pixels were measured and the average was calculated. The S_RGB_ value was then calculated using the following formula, representing the sensitivity as S_RGB_, the total RGB change rate indicating the label [19].

(5) $$\Delta R=|R_{0}-R|;\;\Delta G=|G_{0}-G|;\;\Delta B=|B_{0}-B|\;S_{RGB}=\frac{∆R+∆G+∆B}{R_{0}+G_{0}+B_{0}}\times 100\%$$

where *R*_0_, *G*_0_, and *B*_0_ represent the red, green, and blue values of the label before being exposed to the ammonia solution, respectively; *R*, *G*, and *B* are the values after the reaction.

#### 2.5.4. Indicator Label Color Stability

A colorimeter was used to measure the initial color of the film. Then, the film was stored at 4 °C, 25 °C, and 25 °C under natural light for 12 days. Color values were measured every 2 days. The color difference of the label was calculated according to Formula (2).

#### 2.5.5. Determination of Mechanical Properties

The mechanical properties were determined by referring to the method of Lin et al. [20] with slight modifications. The thickness values of the label were measured with a micrometer. The tensile strength (TS) and elongation at break (EB) of the films were measured by a texture analyzer (TA-XT plus, Stable Micro Systems, Godalming, UK). The initial clip distance is 50 mm and the stretching rate is 0.5 mm/s. TS and EB are calculated according to Formulas (6) and (7), respectively:

(6) $$TS=\frac{F}{M}\times d$$

where TS is tensile strength, MPa; F is the maximum tensile force borne by the specimen when it breaks, N; M is the width of the film, mm; and d indicates the thickness of the label, mm.

(7) $$EB=\frac{L-L_{0}}{L_{0}}\times 100$$

Here, EB is elongation at break, %; L_0_ is the initial length of the film, mm; and L is the final length of the film base tag breaking, mm.

### 2.6. Freshness Indication Label Application

#### 2.6.1. Freshness Indication

Two fresh prawns and 50 g of fresh chicken were placed separately in a food-grade PP preservation box (10 cm × 8 cm). The SP-LB sample (30 mm × 10 mm) was attached to the inner layer of the box lid. The box was then placed in an incubator at 25 °C. A colorimeter was used to measure the colorimetric value of each time period label, and ΔE of the label was calculated according to Formula (2). Photos were taken at regular intervals to record the appearance of the samples and the label.

#### 2.6.2. pH Measurement

Ten grams of shrimp and chicken were grated, respectively, and mixed with 100 mL of KCl solution [21]. The pH value of the filtrate was determined using a precision pH meter (Shanghai Yi Electrical Scientific Instruments Co., Ltd., PHS-25, Shanghai, China).

### 2.7. Data Analysis

All experiments were conducted with a minimum of three replicates. The results were expressed as mean ± standard deviation. Statistical comparison of means among multiple groups was carried out using a one-way analysis of variance (ANOVA) and LSD’s test. Data were analyzed by the SPSS software (version 24.0, SPSS Inc., Chicago, IL, USA). Values of *p* < 0.05 were considered statistically significant.

## 3. Results and Discussion

### 3.1. pH Color Development of Natural Pigments and Visible Spectral Analysis

Due to the production of organic acids, volatile basic nitrogen, CO, and H_2_S, the change in the freshness of meat products is typically accompanied by a change in pH value [14]. This is the principle by which pH labels monitor the freshness of meat products.

**Figure 1:**
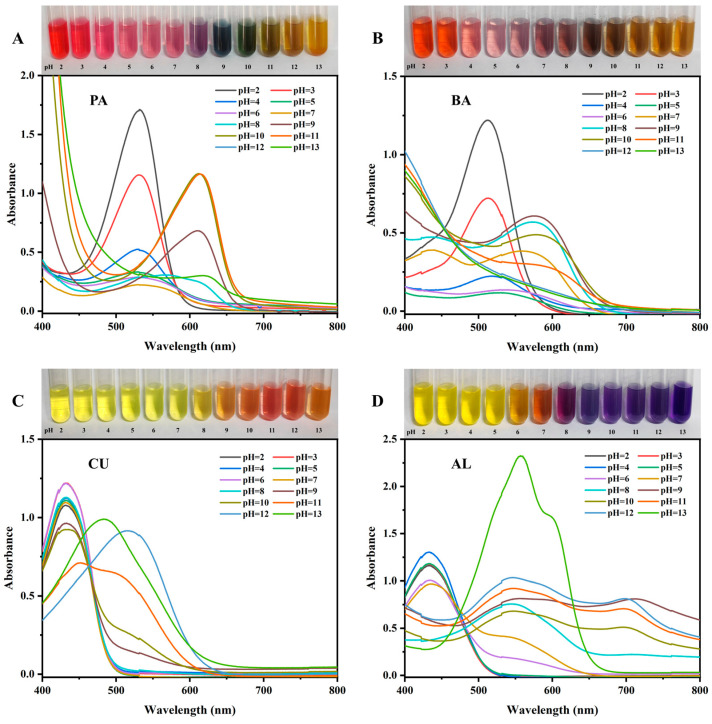
Color development of (**A**) PA, (**B**) BA, (**C**) CU, and (**D**) AL at different pH and ultraviolet–visible spectra.

The structural changes of PA and BA under different pH conditions result in various colors [22]. The results indicated that as the pH ranged from 2 to 13, PA exhibited a color transition from red to purple, indigo blue, cyan, and finally yellow (Figure 1A). The visible spectral data elucidated the mechanism behind the pigment solution’s color change. As the pH value increased from 2 to 13, the maximum absorption peak of PA shifted towards longer wavelengths, resulting in an overall redshift phenomenon, which manifested as the color change in the solution [23]. When the pH was less than 3.0, the BA solution appeared distinctly red. As the pH increased, the red color gradually diminished; when the pH was between 7 and 9, the solution turned blue-purple; with further increases in pH, the BA solution became yellowish-brown (Figure 1B). The corresponding color changes, maximum absorption peak shifts, and variations in intensity in the chromatograms and visible spectral data of BA were caused by changes in the conjugated structure of the anthocyanins [24]. Overall, PA exhibited more color changes and better color development sensitivity than BA. CU appeared yellow in acidic conditions and orange-red in alkaline conditions. This change occurred because pH affected the keto-enol equilibrium of CU, making it pH-sensitive [25]. CU exhibited less color change in the pH range of 2–13, with only two cut-off points at pH 8 and 10 (Figure 1C). Thus, comparatively, CU was not as sensitive as anthocyanin pigments for color development. The visible spectral data showed that as the pH increased, the maximum absorption peak of the CU solution redshifted, resulting in a color change. At pH 8, the AL solution exhibited a distinct purple color; when the pH was greater than 9, the solution displayed an overall blue-violet color (Figure 1D). The color change of the AL solution was also associated with changes in the molecular structure. As the pH increased, the absorption peak of the visible spectrum of AL continuously redshifted, moving from 430.0 nm to 558.0 nm. At pH 2–6, the absorption curve had the maximum absorption peak in the range of 433.0–434.5 nm, when the nitro and azo benzyl groups of the azo phenyl group on the surface of the AL molecule were substituted by the hydroxyl group in the aromatic ring, resulting in a yellow color [26]. When the pH value was greater than 7, due to the resonance effect caused by the deprotonation of the hydroxyl group, which resulted in a redshift, the intensity of the light absorption peaks decreased, and the AL solution produced an obvious color change [27].

In conclusion, all four natural pigments were able to produce color responses to pH changes. Among them, PA had the widest color reaction range and the highest color development sensitivity followed by BA, but its ability to discriminate the color change was not as good as that of PA under the condition of smaller pH change; the color reaction ranges of CU and AL were lower than those of the above two, and there was no obvious color change under acidic condition. Therefore, PA was the best pigment for pH indication.

### 3.2. Effect of Temperature and Light on the Stability of 4 Natural Pigments

**Figure 2:**
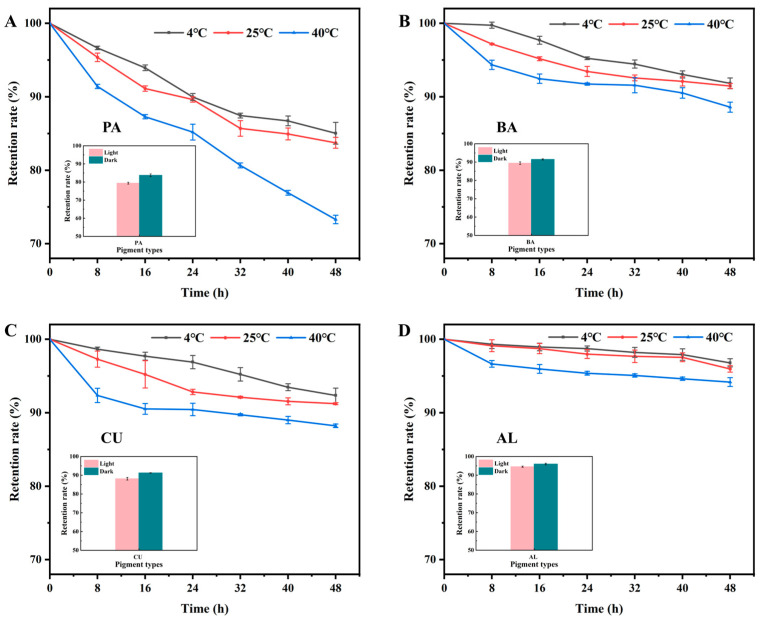
Effect of temperature and light on the stability of four natural pigments: (**A**) PA; (**B**) BA; (**C**) CU; (**D**) AL.

The results of pigment retention at three temperatures showed that the highest pigment retention was found in AL and the lowest in PA at the same temperature, while the pigment retention in CU and AL was similar. After 48 h of storage at 4 °C, the retention rates of the four pigments were 96.79% for AL, 92.35% for CU, 91.83% for BA, and 85.03% for PA. After 48 h of storage at 25 °C, the retention rates of the four pigments were 95.96% for AL, 91.47% for BA, 91.23% for CU, and 83.72% for PA from high to low (Figure 2). After 48 h of storage at 40 °C, the retention rates of the four pigments were AL94.17%, BA88.95%, CU88.21%, and PA73.29%. Among them, the pigment retention rate of PA was most affected by temperature. In particular, the retention of PA at 48 h of storage decreased by 11.74% at 40 °C compared to 4 °C. Temperature had the least effect on the retention of AL. After 48 h of storage at 40 °C, the pigment retention of AL decreased by 2.62% compared with 4 °C.

The results show the pigment retention of the four natural pigments after exposure to light and dark for 48 h at room temperature. Light decreased the retention of the four pigments from high to low by 4.38% for PA, 3.10% for CU, 2.05% for BA, and 1.41% for AL (Figure 2). Thus, the stability of AL was least affected by light and the stability of PA was most affected by light.

In summary, the stability of PA was lower than that of the other three pigments under different conditions, while the stability of AL was the best. In particular, temperature had a greater effect on the stability of the four natural pigments compared to light.

### 3.3. Color Development and Visible Spectral Analysis of Mixed Pigments at Different pH Values

The results of the color sensitivity and stability experiments showed that PA had excellent color sensitivity but poor stability. Although AL’s color sensitivity was moderate, its color change was pronounced within the color development interval, and it had the best stability. Therefore, AL, with the highest stability, was selected to be added to PA, which had the strongest pH color sensitivity. The color sensitivity and stability of the PA-AL mixed pigments in different proportions were further explored to improve the stability of the pigment without compromising its color sensitivity [28].

**Figure 3:**
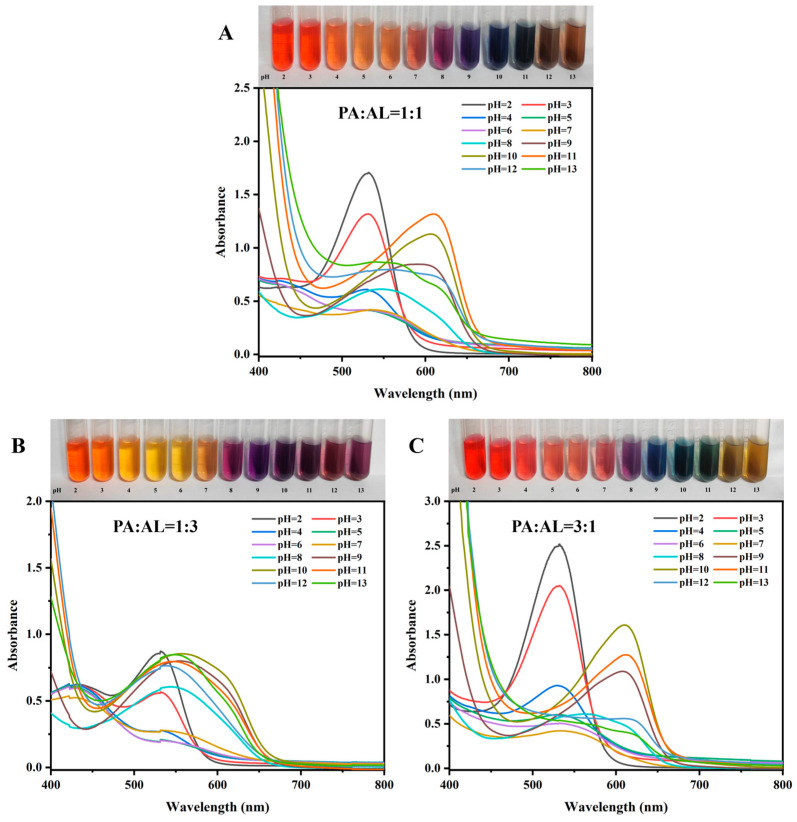
Color development of different ratios of PA-AL mixed pigments. at different pH and ultraviolet–visible spectra. (**A**) PA: AL = 1:1; (**B**) PA: AL = 1:3; and (**C**) PA: AL = 3:1.

The results showed that compared to the single pigment solution, the mixed pigment solution exhibited richer color changes under different pH conditions. When the ratio of PA to AL was 1:1, the solution showed a bright orange-red color. It appeared orange-yellow at pH 4–6, purple-orange at pH 7, and dark purple at pH 8. As the pH increased further, the solution turned blue, blue-green, and brown sequentially (Figure 3A). When the ratio of anthocyanins to AL was 1:3, the color change trend of the solution was similar to that of the single AL solution (Figure 3B). At a ratio of 3:1, the solution showed the most pronounced color changes with varying pH levels. Under strongly acidic conditions, it appeared orange-red and orange-pink. At pH 7, it turned pink-purple, and at pH 8, it was purple. As the pH level increased further, the solution turned blue, turquoise, and yellow-green in sequence (Figure 3C).

The visible spectra results indicated that the spectra of mixed pigment solutions with ratios of 1:1 and 3:1 were similar. The visible spectrum trend was consistent with that of the PA solution in Figure 1A. This suggests that the response of anthocyanins to the visible spectrum was more significant in the mixed solutions, explaining why mixed pigment solutions with ratios of 1:1 and 3:1 exhibited richer color changes under different pH conditions. When the ratio of PA to AL was 1:3, the spectral pattern changed significantly, and the overall maximum absorption peak decreased notably (Figure 3B). Therefore, at a ratio of 1:3, the pH color rendering response of the mixed pigment solution was moderate, and the overall color change was relatively small.

In summary, the color rendering sensitivity of pigments was enhanced by the additive effect of mixed pigments [29]. When the ratio of anthocyanins to AL was 1:1, the pH sensitivity of the mixed pigment was not lower than that of a single PA. At a ratio of 3:1, the color sensitivity of the mixed pigments was higher than that of a single PA.

### 3.4. Effect of Temperature and Light on the Stability of Mixed Natural Pigments in Different Proportions

**Figure 4:**
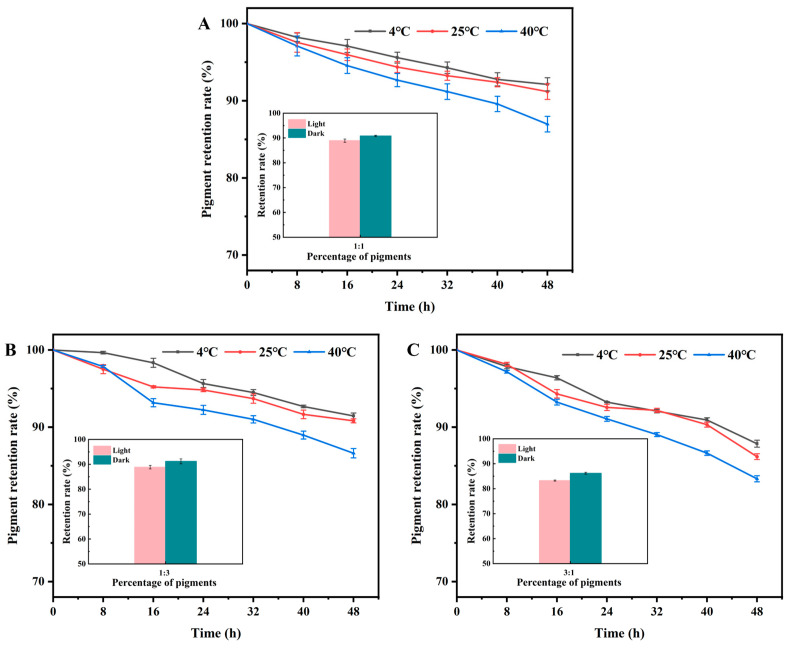
Effect of temperature and light on the stability of mixed natural pigments in different proportions. (**A**) PA:AL = 1:1; (**B**) PA:AL = 1:3; and (**C**) PA: AL = 3:1.

The results showed that the stability of the mixed pigments also decreased with increasing temperature. The highest stability was observed at a temperature of 4 °C, and the stability decreased significantly at 40 °C (Figure 4). However, the stability of the mixed solutions of PA and AL with different ratios was affected by temperature with some differences. After 48 h of storage at 4 °C, the pigment retention rate was 91.46% when the ratio of PA to AL was 1:1, which was 6.16% higher than that of the single PA (Figure 4A). The pigment retention rate was 92.12% when the ratio of PA to AL was 1:3, which was not much different from that of the ratio of 1:1 (Figure 4B). The retention rate was 87.85% when the ratio of PA to AL was 3:1, which was slightly higher than that of a single PA (Figure 4C). After 48 h of storage at 25 °C, the pigment retention rates were 90.82%, 91.18%, and 86.17% for the ratios of PA to AL of 1:1, 1:3, and 3:1, respectively, which were 7.1%, 7.46%, and 2.45% higher than those of single PA, respectively. When stored at an ambient temperature of 40 °C for 48 h, the pigment retention of the three proportions of mixed pigments was 86.62%, 86.96%, and 83.31%, in that order. This was an improvement of 13.33%, 13.67%, and 10.02%, respectively, compared to the single PA. The presence of AL increased the stability of the mixed pigments compared to the single PA. When the ratio of PA to AL was 3:1, the stability of the mixed pigment solution was relatively low due to the large proportion of PA in the system. Therefore, when the mixing ratio of PA and AL is 1:1 and 1:3, the mixed system has better temperature stability. The mixed-use system can buffer the temperature sensitivity of the anthocyanin by the action of AL, thus improving the overall stability.

The results showed that when the ratio of anthocyanins to AL was 1:1, the mixed pigment system had a high pigment retention rate of 88.91% under light conditions and 90.82% under dark conditions (Figure 4A). Compared to the single PA shown in Figure 2A, the retention rates increased by 9.56% and 7.09%, respectively. When the ratio of PA to AL was 1:3, the pigment retention rate increased by 9.49% and 7.45% under light and dark conditions, respectively (Figure 4B). At a ratio of 3:1, the pigment retention rate of the mixed pigments was the lowest, which was still 3.89% and 2.44% higher than that of PA under light and dark conditions, respectively (Figure 4C).

When the ratio of PA to AL was 1:1 and 1:3, the pigment retention rate was higher than at a ratio of 3:1. However, in general, it was higher than that of single PA, indicating that the co-pigments had good stability [30]. At these ratios, anthocyanins and AL may have formed a more stable complex through intermolecular interactions, such as hydrogen bonding and hydrophobic interactions.

The optimal ratio of PA and AL can be determined by combining the stability test results with the color rendering of the mixed pigment. When the ratio of anthocyanins to AL was 1:1, the mixed pigment system exhibited better pH sensitivity and higher stability. Therefore, the combination of purple sweet potato anthocyanin and AL can be used as a natural color development agent for freshness indicator labels, with the optimal ratio being 1:1.

### 3.5. Substrate Screening and Characterization of Indicator Labels

#### 3.5.1. Appearance and Color

Table 1 shows the appearance and color of the six film-based indicator labels. Due to the different ways of interaction between various substrates and the PA-AL mixed pigments, as well as the transparency and light absorption properties of the substrates, the color and appearance of different film indicator labels are also different. There was no significant difference in the L* value of the six indicator labels (*p* < 0.05), indicating that the brightness of the six labels was consistent. The SP appeared brown with a smooth surface and low viscosity. The LB was rosy red, with relatively high viscosity, good toughness, and a smooth surface. The a* value and b* value of the SA were close to those of the SA-LB, making both appear light purple. The addition of sodium alginate reduced the viscosity of the locust bean gum film to some extent. The SP-LB showed a peach-pink color and a uniform surface, indicating that soy protein isolates and locust bean gum combined well. The SP-SA appeared ochre, but its surface was rough and textured.

#### 3.5.2. Water Content and Water Solubility

The water content and water solubility of film labels were affected by the type of film substrate. The results showed that polysaccharides had higher water content and water solubility than proteins (Table 2). This is because polysaccharides contain a large number of hydroxyl groups, which exhibit hydrophilicity, leading to high solubility in water. The LB had the highest moisture content, up to 26.13%. The water content of the SP and the SP-SA was lower, at 14.12% and 12.60%, respectively. For composite labels, hydrogen bonding and electrostatic interactions occurred between polysaccharides (locust bean gum and sodium alginate) and soy protein isolates. The hydrophilic groups of polysaccharides interacted with the hydrophobic groups of proteins, making the film structure denser. This reduced the permeability and retention ability of water molecules, thereby lowering the moisture content of the film labels [31].

Water solubility is an important parameter reflecting the water resistance and shape stability of film-based labels. Increased water solubility may result in partial dissolution of the pigment. Film labels with high water solubility had low water resistance and difficulty maintaining integrity, thus affecting the final indication effect. Therefore, films with low water solubility were more suitable for practical applications [13]. The water solubility of SP and SP-LB was relatively low, while the water solubility of indicator labels containing sodium alginate was higher.

#### 3.5.3. pH and Ammonia Responses

**Figure 5:**
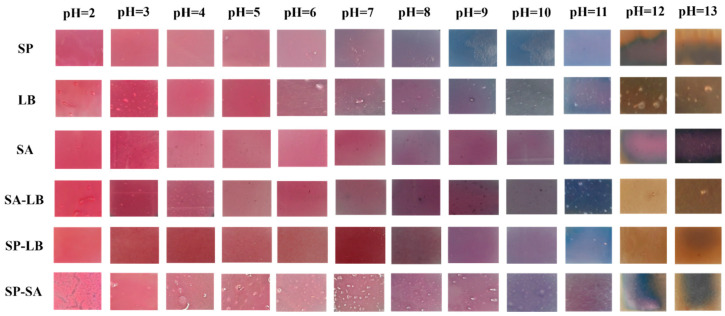
pH response of six film indicator labels.

The results of the pH response showed that when pH < 6, the color of all six labels was red. When the pH value reached 7, the SP, LB, and SA-LB began to show a blue-purple color, and the SP-LB exhibited a distinct wine-red color, while the SA and SP-SA did not change significantly. As the pH increased to 11, the blue color of the SP, SA-LB, and SP-LB gradually deepened, and the purple color of the LB, SA, and SP-SA also intensified. When the pH values reached 12 and 13, all the labels except for the SA and SP-SA exhibited a distinct yellow-brown color (Figure 5). The specific chroma values of the six labels under different pH conditions are shown in Table S1. These results demonstrated that the six labels had good pH response characteristics and could effectively indicate changes in pH. The color change trends of the six indicator labels were similar but not entirely consistent with the color change results of the pigment solution, further indicating that the substrate used to fix the pigment also played a role in the color change of the labels.

**Figure 6:**
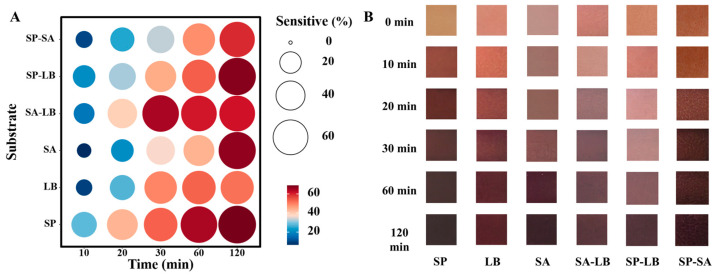
(**A**) Ammonia reaction sensitivity and (**B**) physical diagram of ammonia reaction of six film-based indicator labels.

The response performance of the freshness indicator label to ammonia reflected its sensitivity to alkaline volatile gas detection, which is of great significance for practical applications. The results showed that all six film indicator labels produced obvious color changes after exposure to volatile ammonia. ΔE was greater than 5 (Table S2), with changes that could be identified by the naked eye, and the color changes became more pronounced over time **(**Figure 6B). The color of both the SP and SP-SA labels is less pronounced, deepening from the initial yellowish-brown to brown. The color of the LB and SA-LB darkened with time but did not change much after 20 min. Both SA and SP-LB had more pronounced color changes at all time points from 120 min of ammonia response. However, SP-LB showed a greater range of color changes than SA, which ranged from lavender to purple-brown, and SP-LB, which ranged from pink to lavender to purple-brown. The color changes of the six indicator labels corresponded to the alkaline enhancement resulting from the hydrolysis of ammonia to ammonium ion (NH^4+)^ by steam hydration [32].

Figure 6A visualizes the rate of change of RGB representing the sensitivity of the ammonia response. The size of the circle and the depth of the red color are proportional to the RGB rate of change. The results showed that the three labels that reached the maximum rate of change in RGB at 20 min of ammonia response were SP (43.41%), SA-LB (39.35%), and SP-LB (31.77%). This indicates that these three labels responded most rapidly to ammonia. When the ammonia response was 120 min, the three labels that reached the maximum rate of change in RGB were SP (68.68%), SP-LB (67.34%), and SA (66.59%). It shows that these three labels have the largest span of color change in response to ammonia. SA-LB showed no increase in the rate of RGB change after 30 min. Also, from Table 2, it can be seen that the water solubility of SA-LB is too high and may be dissolved by water vapor when used as a freshness indicator for fresh foods, resulting in indicator bias. The rate of increase in the RGB rate of change of SA was low and is prone to hysteresis in freshness indication applications. In summary, SP and SP-LB have better ammonia responsiveness.

#### 3.5.4. Label Color Stability and Mechanical Property

**Figure 7:**
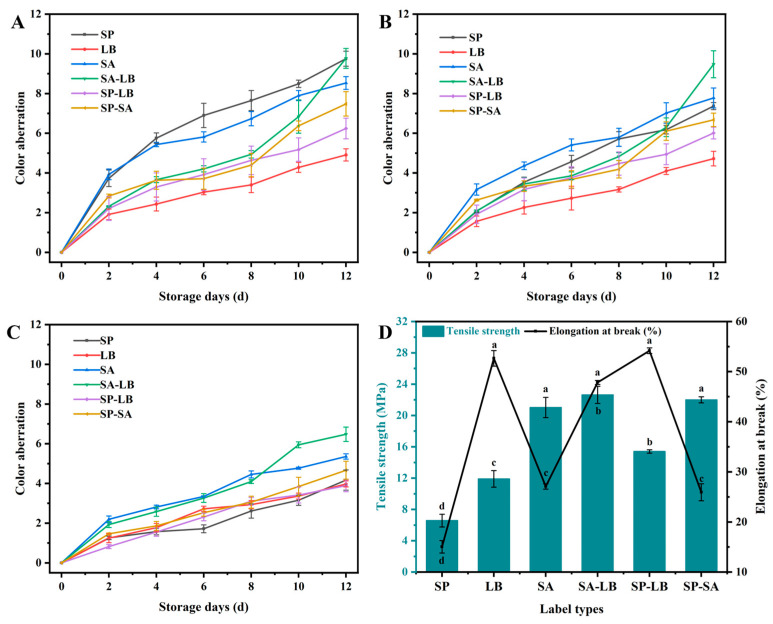
Changes in ∆E values of the six labels when stored for 12 d under different conditions: (**A**) 25 °C, natural light storage; (**B**) 25 °C, dark storage; (**C**) 4 °C, dark storage; and (**D**) these mechanical properties. Different lowercase letters indicate significant differences (*p* < 0.05) in multi-range analyses among the groups.

When the indicator label was used for food freshness monitoring, its color change directly affected the accuracy of the detection results. The application environment of fresh food and fresh indication labels is mostly refrigerated (4 °C) and at room temperature (25 °C). Therefore, it was necessary to compare the influence of light and temperature on the stability of the six film labels by measuring the ΔE of the labels during storage to select the best label substrate. The results showed that the color stability of indicator labels prepared with different substrates varied and was affected by different storage conditions to different degrees (Figure 7). Light exposure increased the ΔE value of the six labels during storage and decreased their stability. This was because light led to changes or decomposition of the pigment structure in the label, promoted the oxidation reaction of the pigment, and generated free radicals, causing the pigment to fade or change color [33]. The smaller the ΔE value, the smaller the color change of the label, indicating that the label is more stable. The results showed that after 12 days of storage at room temperature under dark conditions, the ΔE values of the six labels were LB (4.72), SP-LB (6.01), SP-SA (6.68), SP (7.37), SA (7.78), and SA-LB (9.48) in descending order. Under light conditions, the ΔE values of the six labels increased by LB (0.19), SP-LB (0.23), SA-LB (0.3), SA (0.78), SP-SA (0.8), SP (2.39) (Figure 7). Therefore, the SP was most affected by light, while the stability of the LB and SP-LB was less affected by light. The ∆E values of the six labels were LB, SP-LB, SP-SA, SP, SA, and SA-LB from smallest to largest when stored for 12 days in darkness at 4 °C and in darkness at 25 °C. The ∆E value of SP was the largest when stored for 12 days in light at 25 °C, which indicated that its stability had decreased significantly, whereas the remaining labels had no change in the order of the size of their stability (Figure 7). In summary, compared to other labels, both LB and SP-LB show higher color stability during storage under different conditions.

Determining the mechanical properties of the labels was a key aspect of developing high-quality freshness indication labels. The results showed that the composite of two substrates enhanced the mechanical properties of the labels to a certain extent. For single-substrate labels, the TS of SA and LB were 21.02 MPa and 11.90 MPa, respectively, whereas the TS of SP was only 6.58 MPa. The TS of the three composite labels, SA-LB, SP-SA, and SP-LB, was elevated to 22.64 MPa, 21.99 MPa, and 15.40 MPa higher than that of the single-substrate labels (Figure 7D). The label with the highest TS was SA-LB. This improvement may have been due to the formation of stronger molecular interactions and a composite network structure between proteins and polysaccharides, which enhanced the mechanical properties of the films [34,35]. For single-substrate labels, the EB of LB and SA was 52.65% and 27.09%, respectively. The EB of SP was only 15.05%. The EB of the three composite labels, SA-LB, SP-SA, and SP-LB, was higher than that of the single-substrate labels (Figure 7D). Among the composite labels, the EB of the SP-LB was as high as 54.17%. This may have been because locust bean gum, being a high molecular polysaccharide with high flexibility and good rheological properties, formed a uniform flexible network structure in the film when compounded with soy protein isolate. This network improved the ductility of the film, making it more elastic and flexible under stress. Thus, SA-LB and SP-LB have better performance on TS and EB, respectively.

### 3.6. Application of SP-LB in Freshness Indication of Shrimp and Chicken

In conjunction with the above metrics related to label characterization, the substrates of the labels were screened for better application in shrimp and chicken freshness indication. Through the measurement and comparison of six labels, the following conclusions were drawn: In terms of water content, water solubility, pH, and ammonia response, SP and SP-LB performed best. In terms of color stability, LB and SP-LB performed better. In terms of mechanical properties, SA-LB and SP-LB performed better. Therefore, after comprehensive evaluation and analysis, the combination of soy protein isolates and locust bean gum was selected as the best label substrate.

The high water content and neutral pH value of aquatic products facilitate the degradation of proteins by endogenous enzymes, providing an ideal medium for microbial growth [36]. In the food industry, monitoring the spoilage process of chicken meat is a crucial cornerstone to ensure product quality and nutritional safety [37]. Therefore, using pH-sensitive indicator labels for freshness monitoring of shrimp and chicken is of great significance. Table 3 shows the application of the SP-LB to shrimp and chicken freshness indicators. At room temperature, fresh products are susceptible to microbial contamination, leading to an increase in alkaline compounds and a corresponding rise in pH value during storage [38,39]. The pH of shrimp and chicken increased with storage time. The color of SP-LB changed over time, with specific color parameters shown in Table S3. The color change of the label used to indicate the freshness of shrimp generally appeared from lavender to dark purple, then blue-purple, and finally gray. When the shrimp was stored for 9 h, the label appeared purple, and when the pH value was 7.08, ΔE reached 13.84, showing a noticeable difference from the initial color of the label to the naked eye. When the storage time reached 15 h, the pH value of the shrimp was 7.23, indicating deterioration [21], and the label edge showed an obvious blue-purple color. After more than 15 h of storage, the shrimp had completely spoiled, and the label turned completely blue-purple. The label color change used to indicate the freshness of the chicken appeared from lavender to purple with a green edge, and finally to bluish-purple. When the chicken was stored for 24 h, the pH reached 6.76, and the label showed an obvious purple color, indicating that the chicken had deteriorated. When the storage time reached 28 h, the pH rose to 7.04, ΔE reached as high as 21.25, and the label edge appeared green. As the pH of the chicken continued to rise, the label appeared blue-purple, indicating that the chicken had spoiled. The experimental results showed that the prepared SP-LB could effectively indicate the deterioration of protein food. Further research will be conducted to further optimize the labeling system so that the color change of SP-LB can accurately reflect the freshness of the product.

## 4. Conclusions

In this study, the pH color development properties, UV–visible spectra, and temperature and light stability of natural pigments were determined. It was found that purple yam anthocyanin had excellent color development properties and, when mixed with AL at a ratio of 1:1, further enhanced pigment stability and sensitivity. Film labels were prepared using three natural biomacromolecules: soy protein isolates, acacia bean gum, and sodium alginate. By comparing ammonia response sensitivity, color stability, water solubility, and mechanical properties, the soy protein isolate and acacia bean gum composite were identified as the best substrate system. The results showed that the SP-LB exhibited excellent performance in terms of pH and ammonia response sensitivity, as well as color stability. Additionally, preliminary validation tests demonstrated that the label showed significant application potential in the freshness indication of shrimp and chicken. Future studies can further optimize the combination of pigment and substrate, prepare different forms of indicator labels, improve their performance, explore the impact of initial pH value on indicator performance, conduct a comprehensive determination of protein food freshness indicators, and further enhance the correlation between label color and food freshness. This will facilitate the development of labels that can accurately distinguish the freshness of various aquatic and meat products to meet the higher application requirements of the food industry.

## Figures and Tables

**Table 1:** Color parameters of film-based indicator label systems with different substrate materials.

Labels	L*	a*	b*	ΔE	Appearance
SP	62.83 ± 0.47 ^d^	21.93 ± 0.23 ^a^	28.27 ± 0.45 ^a^	52.03 ± 0.15 ^a^	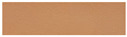
LB	69.83 ± 0.49 ^c^	19.43 ± 1.01 ^b^	12.10 ± 0.44 ^d^	36.95 ± 0.58 ^c^	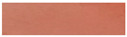
SA	71.47 ± 1.01 ^b^	19.63 ± 0.78 ^b^	7.67 ± 0.31 ^f^	33.98 ± 1.29 ^d^	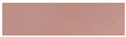
SA-LB	69.67 ± 0.47 ^c^	20.20 ± 0.62 ^b^	9.00 ± 0.46 ^e^	36.14 ± 0.57 ^c^	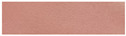
SP-LB	72.70 ± 0.44 ^a^	14.37 ± 1.15 ^d^	18.97 ± 0.76 ^b^	36.60 ± 1.02 ^c^	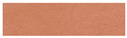
SP-SA	69.03 ± 0.47 ^c^	16.80 ± 0.26 ^c^	17.47 ± 0.15 ^c^	39.13 ± 0.36 ^b^	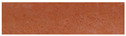

Note: different lowercase letters indicate significant differences (*p* < 0.05) in multi-range analyses among the groups.

**Table 2:** Water content and water solubility of film-based indicator labels for different substrate materials.

Labels	Water Content (%)	Water Solubility (%)
SP	14.12 ± 1.44 ^cd^	46.71 ± 4.12 ^d^
LB	26.13 ± 3.24 ^a^	56.97 ± 3.77 ^bc^
SA	15.94 ± 3.60 ^bcd^	77.77 ± 6.23 ^a^
SA-LB	18.23 ± 2.28 ^bc^	77.65 ± 2.09 ^a^
SP-LB	19.14 ± 0.99 ^b^	51.50 ± 2.11 ^cd^
SP-SA	12.60 ± 1.26 ^d^	62.28 ± 5.77 ^b^

Note: different lowercase letters indicate significant differences (*p* < 0.05) in multi-range analyses among the groups.

**Table 3:** Changes in pH of shrimp and chicken at 25 °C and color response of SP-LB.

Shrimp					Chicken Breast				
Time (h)	pH Value	Shrimp Freshness Indicator Physical Picture	Color Change of SP-LB	ΔE	Time (h)	pH Value	Shrimp Freshness Indicator Physical Picture	Color Change of SP-LB	ΔE
0	6.66 ± 0.02 ^i^	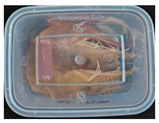	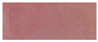	-	0	5.61 ± 0.03 ^i^	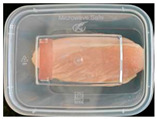	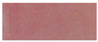	-
3	6.88 ± 0.01 ^h^	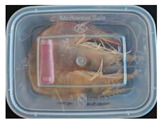	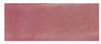	2.79 ± 0.53 g	4	5.70 ± 0.03 ^h^	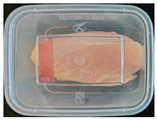	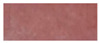	3.56 ± 0.72 ^f^
6	6.98 ± 0.02 ^g^	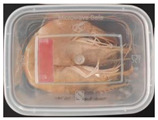	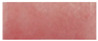	6.53 ± 0.53 f	8	5.77 ± 0.03 ^g^	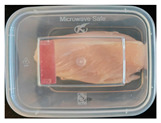	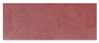	4.43 ± 0.74 ^f^
9	7.08 ± 0.01 ^f^	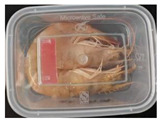	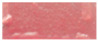	13.84 ± 0.42 e	12	6.14 ± 0.03 ^f^	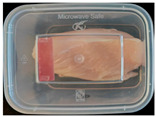	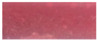	12.86 ± 1.56 ^e^
12	7.15 ± 0.01 ^e^	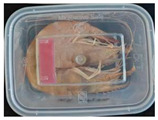	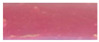	15.96 ± 1.59 d	16	6.30 ± 0.02 ^e^	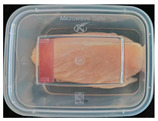	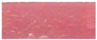	16.4 ± 0.84 ^d^
15	7.23 ± 0.02 ^d^	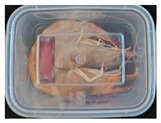	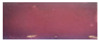	17.75 ± 0.61 d	20	6.57 ± 0.03 ^d^	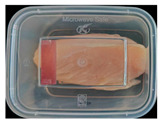	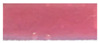	19.08 ± 1.16 ^c^
18	7.41 ± 0.02 ^c^	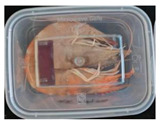	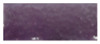	25.27 ± 1.42 c	24	6.76 ± 0.02 ^c^	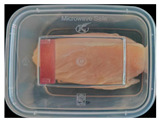	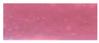	19.20 ± 1.18 ^c^
21	7.57 ± 0.02 ^b^	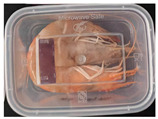	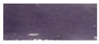	29.4 ± 1.76 b	28	7.04 ± 0.03 ^b^	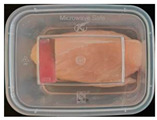	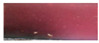	21.25 ± 0.88 ^b^
24	7.66 ± 0.02 ^a^	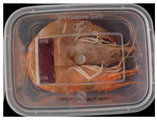	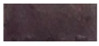	31.5 ± 0.65 a	32	7.32 ± 0.02 ^a^	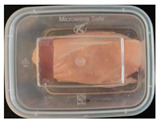	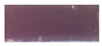	23.68 ± 0.87 ^a^

Note: different lowercase letters indicate significant differences (*p* < 0.05) in multi-range analyses among the groups; “-” indicates that the label at the initial time has no color difference compared to itself.

## Data Availability

The original contributions presented in the study are included in the article; further inquiries can be directed to the corresponding author.

## Supplementary Materials

The following supporting information can be downloaded at: https://www.mdpi.com/article/10.3390/foods13244049/s1, Table S1: Color parameters of the film-based indicator labels under different pH conditions; Table S2: The colorimetric value of the film indicator label changes in response to ammonia; Table S3: The SP-LB colorimetric value for the freshness indication of shrimp and chicken at 25 °C.
