# Supplementary material for: Preparation and Characterization of Bio-Based Freshness Indicator Labels Loaded with Natural Pigments with High Stability and Sensitivity
Source: Foods. 2024 Dec 15;13(24):4049. doi: 10.3390/foods13244049 (PMC11727871; doi:10.3390/foods13244049)
Supplement: Supplementary file 1 [file foods-13-04049-s001.zip › foods-3315084-supplementary.pdf]

Table S1. Color parameters of the film-based indicator labels under different pH conditions.

Labels	pH	*L*	*a*	*b*	△E
SP	2	48.21±1.34[d]	40.18±1.18[cd]	1.73±1.05[cd]	35.41±0.52[ef]
	3	53.36±0.46[bc]	35.54±0.54[b]	4.76±0.05[b]	28.77±0.39[g]
	4	59.20±0.83[a]	23.73±2.18[bc]	3.24±0.53[bc]	25.42±0.76[h]
	5	54.20±0.50[b]	26.43±1.32[d]	1.06±0.98[d]	28.92±1.19[g]
	6	60.36±0.67[a]	19.79±1.02[e]	-2.80±1.51[e]	31.26±1.47[g]
	7	51.22±2.99[c]	12.71±1.52[f]	-6.10±0.52[f]	37.51±1.68[e]
	8	47.07±1.06[de]	8.42±2.06[g]	-10.95±0.65[g]	44.41±0.96[c]
	9	44.31±3.66[ef]	-0.04±1.05[h]	-19.78±1.61[h]	56.06±2.13[a]
	10	42.76±1.29[fg]	0.21±0.67[h]	-19.37±0.40[h]	56.08±0.37[a]
	11	58.00±0.14[a]	5.83±0.92[h]	-20.30±0.56[h]	51.40±0.65[b]
	12	37.54±1.09[h]	4.83±0.92[cd]	1.55±0.71[cd]	40.57±1.50[d]
	13	39.96±1.89[gh]	2.25±0.73[a]	10.83±2.41[a]	34.87±2.86[f]
LB	2	53.56±2.54[a]	37.51±2.50[b]	8.90±2.31[c]	24.64±3.24[fg]
	3	44.69±0.36[d]	42.56±1.03[a]	6.49±0.70[d]	34.63±0.86[cd]
	4	50.93±0.40[ab]	37.96±1.68[b]	3.15±0.67[e]	27.97±1.03[e]
	5	44.90±0.60[cd]	38.50±0.50[b]	4.85±0.17[de]	32.21±0.74[d]
	6	51.31±2.40[ab]	21.81±1.19[c]	-1.02±0.56[f]	22.88±1.88[g]
	7	48.10±2.54[bc]	16.30±4.87[d]	-4.00±0.82[g]	27.56±1.91[ef]
	8	43.63±1.89[d]	17.15±0.65[d]	-10.04±0.15[h]	34.39±1.54[cd]
	9	42.19±2.48[d]	10.44±2.00[e]	-12.80±0.77[i]	38.33±1.91[ab]
	10	48.33±2.13[b]	0.87±0.27[g]	-5.53±0.39[g]	33.47±1.04[cd]
	11	49.95±2.83[b]	5.98±0.75[f]	-15.50±1.67[j]	36.60±2.99[abc]
	12	32.74±0.65[f]	7.63±2.59[ef]	16.73±0.43[a]	39.24±1.38[a]
	13	37.99±1.33[e]	4.23±1.46[fg]	14.85±1.48[b]	35.42±1.70[bcd]
SA	2	46.64±0.85[b]	46.45±1.73[a]	9.32±0.66[a]	36.59±1.80[bc]
	3	45.31±2.59[bc]	45.16±0.84[a]	6.07±0.67[b]	36.60±2.47[bc]
	4	56.29±2.25[a]	29.70±3.61[b]	1.74±0.31[c]	19.22±3.78[f]
	5	53.18±0.51[a]	27.28±0.55[bc]	2.11±0.29[c]	20.60±0.63[f]
	6	55.19±2.09[a]	30.50±2.76[b]	0.30±0.95[d]	21.00±2.78[f]
	7	44.39±3.03[bc]	29.40±4.77[b]	-0.03±0.17[d]	29.94±4.45[de]
	8	42.60±0.93[c]	17.27±3.02[d]	-9.81±1.03[g]	33.93±1.08[cd]
	9	37.83±1.33[d]	23.96±0.98[c]	-8.56±0.56[fg]	37.61±1.50[bc]
	10	45.04±3.26[bc]	12.94±3.44[def]	-7.95±1.73[f]	31.61±2.73[d]
	11	38.65±0.67[d]	10.76±0.96[f]	-13.35±0.68[h]	39.98±1.06[b]
	12	47.79±0.26[b]	15.33±0.19[de]	-5.12±0.92[e]	27.26±0.60[e]
	13	25.84±1.45[e]	11.06±1.60[ef]	-6.36±0.88[e]	48.51±1.75[a]
SA-LB	2	46.26±1.02[c]	49.21±0.61[a]	12.13±1.35[c]	37.43±0.94[bc]
	3	36.91±2.36[d]	39.75±0.62[b]	5.47±0.90[d]	38.33±2.25[bc]
	4	44.79±0.35[c]	32.51±0.46[c]	1.67±0.31[de]	28.71±0.11[d]
	5	50.79±1.29[b]	22.13±1.83[d]	4.96±0.60[d]	19.46±1.51[e]
	6	44.36±2.54[c]	32.58±1.98[c]	3.47±0.59[d]	28.73±3.14[d]
	7	43.06±2.00[c]	16.91±3.90[e]	-1.09±0.14[e]	28.85±1.31[d]

	8	32.64±0.30^{e}	18.12±0.82^{e}	-7.77±0.49^{fg}	40.71±0.26^{b}
	9	30.39±0.47^{e}	17.17±1.42^{e}	-6.29±0.52^{f}	42.28±0.37^{b}
	10	37.33±4.63^{d}	5.67±1.78^{f}	-10.74±8.29^{g}	40.75±8.47^{b}
	11	30.14±1.14^{e}	4.9±0.78^{f}	-19.83±1.09^{h}	51.28±0.60^{a}
	12	60.29±1.51^{a}	6.38±1.25^{f}	29.06±0.47^{a}	26.15±0.56^{d}
	13	38.35±1.1^{d}	7.75±0.88^{f}	19.13±0.68^{b}	35.21±0.58^{c}
	2	52.42±1.62^{a}	41.47±1.68^{a}	12.09±3.37^{b}	34.67±1.68^{bcde}
	3	41.70±2.04^{abc}	34.81±0.54^{ab}	12.62±0.78^{b}	37.69±1.84^{bcd}
	4	38.36±0.87^{bc}	34.21±0.74^{ab}	12.96±1.11^{b}	40.13±0.35^{abc}
	5	45.37±1.86^{abc}	28.42±1.24^{bc}	9.38±0.42^{b}	32.21±1.99^{cde}
	6	46.54±1.96^{abc}	23.99±2.92^{cde}	9.97±0.71^{b}	29.37±2.47^{de}
SP-LB	7	40.74±20.93^{abc}	25.23±15.65^{cd}	8.81±5.92^{b}	39.20±16.66^{abcd}
	8	35.39±0.32^{c}	15.49±1.74^{ef}	-1.26±0.50^{c}	42.48±0.44^{ab}
	9	44.22±4.20^{abc}	18.92±2.18^{def}	-10.92±1.18^{d}	41.61±3.82^{abc}
	10	48.40±1.09^{ab}	12.47±1.71^{f}	-13.04±0.81^{d}	40.26±1.21^{abc}
	11	49.90±2.30^{ab}	3.14±1.95^{g}	-21.53±1.81^{e}	47.84±3.01^{a}
	12	47.54±0.92^{abc}	12.89±0.46^{f}	29.14±0.43^{a}	27.18±0.73^{e}
	13	38.26±3.55^{bc}	15.43±1.36^{ef}	27.53±5.32^{a}	35.85±2.42^{bcde}
	2	58.77±1.83^{b}	41.11±2.06^{a}	3.01±1.10^{bc}	30.10±2.76^{d}
	3	58.07±0.97^{bc}	35.85±1.77^{b}	7.09±0.61^{a}	24.31±2.02^{e}
	4	57.15±0.45^{bcd}	26.33±1.85^{c}	4.70±0.87^{b}	19.93±1.07^{f}
	5	55.73±1.09^{bcd}	26.37±1.95^{c}	4.53±0.40^{b}	20.92±1.59^{ef}
	6	63.37±0.90^{a}	20.39±0.46^{d}	2.52±0.78^{c}	16.41±0.55^{g}
SP-SA	7	55.03±2.55^{cde}	20.21±3.41^{d}	1.40±0.67^{c}	21.76±2.69^{ef}
	8	54.05±4.48^{de}	20.45±2.57^{d}	-6.15±0.81^{e}	28.41±3.19^{d}
	9	51.60±1.97^{e}	19.16±0.81^{d}	-5.79±0.10^{e}	29.20±1.20^{d}
	10	48.16±1.31^{f}	10.17±0.41^{f}	-14.83±0.94^{f}	39.03±1.42^{b}
	11	43.35±1.30^{g}	14.68±1.64^{e}	-6.84±0.71^{e}	35.46±1.10^{c}
	12	32.53±0.82^{i}	-0.37±1.44^{g}	-14.01±2.55^{f}	51.20±1.99^{a}
	13	36.53±2.19^{h}	0.63±0.76^{g}	-0.88±1.39^{a}	40.70±2.00^{b}

Note: different lowercase letters indicate significant differences ($p < 0.05$) in multi-range analyses among the groups.

Table S2. The colorimetric value of the film indicator label changes in response to ammonia.

Labels	Time (min)	*R*	*G*	*B*	S_{RGB} (%)
SP	0	122.33±4.93^{a}	60.33±4.16^{a}	32.33±1.53^{a}	-
	10	90.00±4.58^{b}	39.00±2^{b}	26.00±1.00^{b}	27.91±3.51^{d}
	20	65.00±6.08^{c}	30.33±3.06^{c}	26.33±2.08^{b}	43.41±5.10^{c}
	30	52.67±8.08^{d}	25.67±4.73^{cd}	22.67±3.79^{bc}	53.02±7.65^{b}
	60	36.33±2.08^{e}	21.33±1.15^{de}	19.33±2.52^{c}	64.19±2.13^{a}
	120	28.67±2.52^{e}	18.33±2.08^{e}	20.33±4.62^{c}	68.68±4.17^{a}
LB	0	142.67±9.29^{a}	78.67±8.08^{a}	54.67±6.11^{a}	-
	10	135.00±8.00^{a}	67.67±3.51^{b}	48.00±1.73^{a}	9.26±4.62^{c}
	20	101.33±11.24^{b}	52.33±5.13^{c}	47.67±6.03^{a}	27.05±7.97^{b}
	30	67.67±8.08^{c}	34.33±3.21^{d}	38.33±4.16^{b}	49.15±5.55^{a}
	60	67.33±2.89^{c}	28.67±1.15^{d}	34.33±2.08^{b}	52.78±2.00^{a}
	120	75.00±8.72^{c}	26.33±1.53^{d}	32.67±2.08^{b}	51.45±3.46^{a}
SA	0	144.33±5.03^{a}	85.00±5.29^{a}	61.00±5.29^{a}	-
	10	139.00±8.00^{a}	78.33±6.51^{a}	61.67±7.37^{a}	6.58±3.67^{e}
	20	108.00±4.00^{b}	63.67±3.06^{b}	58.0±3.61^{a}	21.13±3.29^{d}
	30	87.00±4.36^{c}	49.00±3.00^{c}	48.00±2.65^{b}	36.62±3.29^{c}
	60	71.67±3.21^{d}	44.00±1.73^{c}	47.67±3.06^{b}	43.74±1.63^{b}
	120	42.00±2.00^{e}	25.00±2.00^{d}	30.00±1.00^{c}	66.59±1.50^{a}
SA-LB	0	132.00±2.65^{a}	83.00±1.00^{a}	62.00±2.00^{a}	-
	10	114.67±4.93^{b}	64.00±4.00^{b}	50.67±4.16^{b}	17.21±4.57^{c}
	20	72.00±7.21^{c}	48.00±3.46^{c}	48.00±1.00^{b}	39.35±3.80^{b}
	30	40.67±2.52^{e}	27.67±1.53^{d}	30.67±0.58^{c}	64.26±1.57^{a}
	60	51.33±3.21^{d}	25.67±2.08^{d}	32.00±4.58^{c}	60.65±3.56^{a}
	120	52.00±3.61^{d}	26.33±3.06^{d}	29.67±2.52^{c}	61.01±3.31^{a}
SP-LB	0	137.33±2.89^{a}	77.67±4.51^{a}	48.33±4.04^{a}	-
	10	113.33±8.02^{b}	53.67±7.37^{b}	41.00±3.61^{b}	21.01±7.03^{d}
	20	90.67±6.11^{c}	46.67±6.11^{b}	42.33±5.03^{ab}	31.77±6.19^{c}
	30	73.00±5.57^{d}	37.33±6.03^{c}	35.33±3.79^{bc}	44.68±5.69^{b}
	60	62.00±1.73^{d}	29.00±1.73^{cd}	32.00±2.65^{cd}	53.29±0.38^{b}
	120	37.67±12.34^{e}	22.00±1.00^{d}	26.33±3.06^{d}	67.34±5.14^{a}
SP-SA	0	130.67±5.51^{a}	71.67±3.51^{a}	43.00±2.65^{ab}	-
	10	126.33±8.39^{a}	73.33±8.14^{a}	53.33±7.37^{a}	10.19±2.03^{c}
	20	92.00±13.86^{b}	54.67±8.08^{b}	44.67±7.23^{ab}	24.73±7.18^{b}
	30	79.00±9.00^{b}	45.33±4.16^{b}	42.33±4.51^{b}	33.15±5.51^{b}
	60	58.33±11.02^{c}	34.67±6.66^{c}	34.00±7.00^{bc}	48.23±9.89^{a}
	120	42.33±1.15^{c}	29.00±2.00^{c}	28.67±2.52^{c}	59.24±2.27^{a}

Note: different lowercase letters indicate significant differences ($p < 0.05$) in multi-range analyses among the groups. "-" indicates that the label at the initial time has no color difference compared to itself.

Table S3. The SP-LB colorimetric value for the freshness indication of shrimp and chicken at 25℃

Shrimp				Chicken breast			
**Time (h)**	***L***	***a***	***b***	**Time (h)**	***L***	***a***	***b***
0	52.49±0.92^{bc}	17.70±1.08^{f}	6.20±0.26^{b}	0	52.76±1.34^{a}	16.66±1.67^{d}	7.17±1.01^{a}
3	51.39±0.67^{c}	19.78±0.45^{e}	4.83±0.43^{b}	4	49.64±0.8^{a}	17.48±0.49^{d}	8.54±0.44^{a}
6	54.42±1.44^{ab}	21.81±0.74^{d}	10.71±0.38^{a}	8	48.60±0.81^{a}	17.68±0.69^{d}	7.56±1.05^{a}
9	55.75±0.82^{a}	30.03±0.58^{b}	11.52±0.26^{a}	12	50.60±4.96^{a}	28.49±2.07^{c}	5.46±0.41^{b}
12	40.78±0.64^{e}	26.93±1.50^{c}	1.51±3.94^{c}	16	50.78±6.18^{a}	32.13±0.81^{b}	7.06±0.43^{ab}
15	47.78±0.31^{d}	34.65±0.58^{a}	4.17±1.44^{b}	20	53.10±1.21^{a}	33.83±0.75^{ab}	-1.03±1.32^{d}
18	34.35±2.53^{f}	10.66±1.08^{g}	-9.77±1.13^{e}	24	50.80±0.98^{a}	35.40±1.06^{a}	3.69±1.29^{c}
21	30.12±2.81^{g}	11.22±1.91^{g}	-11.57±0.63^{e}	28	41.35±0.66^{b}	34.48±0.60^{a}	5.42±0.97^{b}
24	24.31±0.71^{h}	6.32±0.37^{h}	-2.08±0.25^{d}	32	32.80±0.93^{c}	12.17±1.61^{e}	-4.64±0.78^{e}

Note: different lowercase letters indicate significant differences ($p < 0.05$) in multi-range analyses among the groups.
